# Quantitative Threshold Determination of Auditory Brainstem Responses in Mouse Models

**DOI:** 10.3390/ijms241411393

**Published:** 2023-07-13

**Authors:** Kenji Tanaka, Shuma Ohara, Tadaaki Matsuzaka, Aira Matsugaki, Takuya Ishimoto, Ryosuke Ozasa, Yukiko Kuroda, Koichi Matsuo, Takayoshi Nakano

**Affiliations:** 1 Division of Materials and Manufacturing Science, Graduate School of Engineering, Osaka University, Suita 565-0871, Japan; 2 Laboratory of Cell and Tissue Biology, Keio University School of Medicine, Tokyo 160-8582, Japan

**Keywords:** auditory brainstem responses (ABR), auditory function, ABR threshold, correlation coefficient, cross-covariance

## Abstract

The auditory brainstem response (ABR) is a scalp recording of potentials produced by sound stimulation, and is commonly used as an indicator of auditory function. However, the ABR threshold, which is the lowest audible sound pressure, cannot be objectively determined since it is determined visually using a measurer, and this has been a problem for several decades. Although various algorithms have been developed to objectively determine ABR thresholds, they remain lacking in terms of accuracy, efficiency, and convenience. Accordingly, we proposed an improved algorithm based on the mutual covariance at adjacent sound pressure levels. An ideal ABR waveform with clearly defined waves I–V was created; moreover, using this waveform as a standard template, the experimentally obtained ABR waveform was inspected for disturbances based on mutual covariance. The ABR testing was repeated if the value was below the established cross-covariance reference value. Our proposed method allowed more efficient objective determination of ABR thresholds and a smaller burden on experimental animals.

## 1. Introduction

The auditory brainstem response (ABR) is an electroencephalogram (EEG) recording obtained from the auditory nervous system between the cochlea and brain during sound stimulation as potential fluctuations on the scalp. In rodents and cats, five representative peaks have been identified in the EEG during sound stimulation. They have been assigned as waves I–V in order of decreasing latency and arise from the auditory nerve, cochlear nucleus, superior olive complex, lateral lemniscal nuclei, and inferior colliculus, respectively [1,2]. The sound pressure level of the sound stimulus is decreased, the latency of each peak increases, its amplitude decreases, and all peaks disappear after a certain sound pressure level. At this sound pressure level, which is termed the ABR threshold (auditory threshold), brain waves generated by the auditory nervous system are not observed. ABR thresholds are commonly used as non-invasive quantitative measures of auditory function. Hearing loss associated with aging and disease has been shown to exhibit a high ABR threshold in genetically modified animals [3,4].

Although ABR testing is widely used to assess auditory function, ABR thresholds are subjective since they are determined visually by humans, which may result in bias [5]. Furthermore, in case of high background activity and system noise, individual differences in skill levels can cause errors [5]. This ambiguity in threshold judgments has been known for several decades [5,6]. Accordingly, various methods have been devised to solve this problem, including computing the cross-covariance using templates [7,8], supervised classification methods using machine learning [9], detection methods using signal-to-noise ratios [10], and methods using neurophysiological parameters, such as the neuron firing rate [11]. Moreover, they have been attempts to automate the procedure.

In the current situation, this paper focuses on a method that calculates cross-covariance between adjacent sound pressure levels [12]. This method calculates the cross-covariance between the ABR waveform and the ABR waveform at one higher sound pressure level, generates a correlation coefficient vs. sound pressure level function from the results, and fits it with a sigmoid or binomial power function. The ABR threshold was determined from an approximate curve according to a pre-determined interphase reference value. This is easy to understand since it follows the same principle as the conventional visual method of determining the threshold values. Additionally, selecting an appropriate threshold value can improve the reliability of comparisons between laboratories and researchers. However, this proposed approach can be further improved. We propose the following two points for improvement:

i. The approximate curve of the correlation coefficient versus the sound pressure level function should be unified with only the sigmoid function;
ii. Instead of judging whether sigmoid function fitting was possible based on criterion *a* < criterion < *b*, 0.005 < *d* < 0.999 (Equation (1)) after fitting, the cross-covariance with the ideal ABR waveforms (template) was calculated as soon as the ABR waveforms were obtained.

(1) $$y=a+\frac{\left(b-a\right)}{1+10^{d(c-x)}}$$

*x* = lower level of correlated pairs (in dB SPL); *y* = correlation coefficient;
*a* = minimum value; *b* = maximum value;
*c* = *x* value at midpoint; *d* = slope.

Based on the aforementioned improvements, this study aimed to establish a simpler and more useful method of determining threshold values by calculating the cross-covariance between adjacent sound pressure levels.

## 2. Results

### 2.1. Ideal ABR Waveform as a Standard Template

The ABR was recorded using recording electrodes placed under the right and left auricularis and a reference electrode placed at the parietal lobe. However, in some cases, a clear waveform could not be obtained due to high-level background activity and system noise, even after 500 additive averaging runs at each sound pressure level. A distorted ABR waveform results in a correlation coefficient level function with a distorted shape; accordingly, sigmoidal function fitting cannot be correctly performed. Therefore, a method of discriminating between disturbed ABR waveforms is essential.

**Figure 1:**
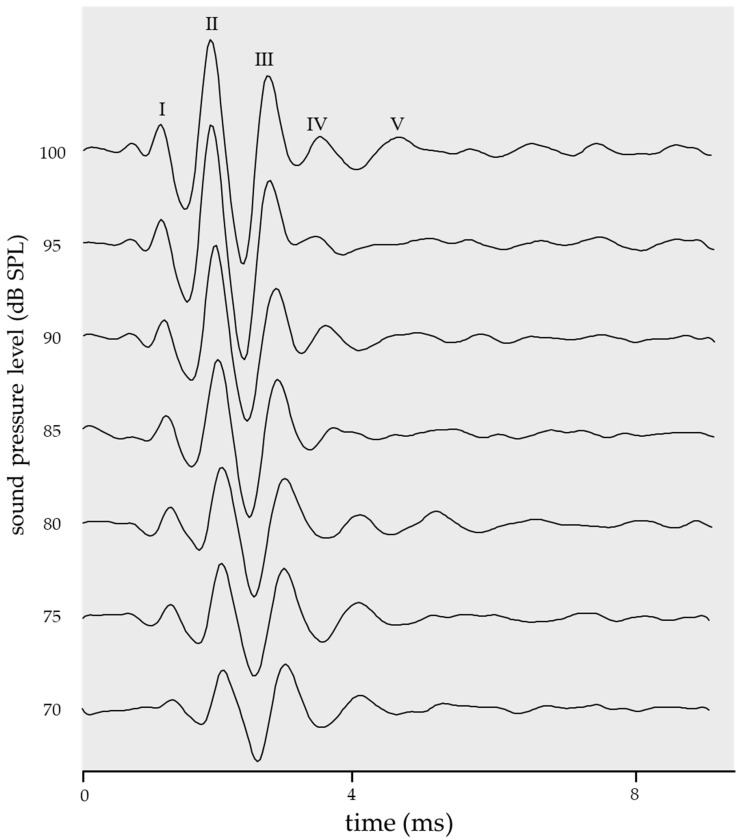
The ideal ABR waveform (standard template) was created to determine if the ABR waveform is suitable for sigmoid function fitting. It was created by averaging ABR waveforms (WT: n = 7), for which sigmoid function fitting was successfully performed. The calculated cross-covariance range was set to 70–100 dB SPL because waves I–V are clearly identified within this sound pressure range in mice.

Accordingly, we established the ideal ABR waveform (standard template) (Figure 1) by averaging the ABR waveforms (n = 7), for which sigmoid function fitting was successfully performed. The cross-covariance between the template and obtained waveforms was calculated. When the average correlation coefficient for the seven pressure levels between 70 and 100 dB SPL (in 5-dB SPL increments) was <0.60, the ABR waveform was determined to be disturbed. The calculated range of cross-covariance was 70–100 dB SPL since waves I–V were clearly identified within this range in wild-type (WT) mice. Further, the electrode positions were readjusted, and ABR testing was repeated in case of a disturbed ABR waveform, which was unsuitable for sigmoid function fitting.

### 2.2. Threshold in the Cross-Covariance with a Standard Template

A single reference value was required to determine whether the waveform was disturbed based on the correlation coefficient with the ideal ABR waveform (standard template). Therefore, we calculated the root-mean-square error (*RMSE*) of the sigmoidal approximation curve and evaluated the value of the correlation coefficient when the *RMSE* increased. The *RMSE* was calculated using Equation (2):

(2) $$RMSE=\sqrt{\frac{1}{n}\sum_{i=0}^{n-1}{\left(x_{i}-y_{i}\right)}^{2}}$$

*x* = observed values; *y* = expected values; and *n* = sample size.

**Figure 2:**
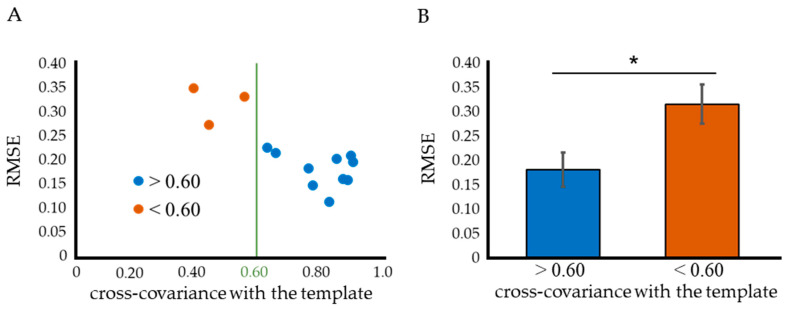
A reference value for determining if a waveform is suitable for sigmoid function fitting based on its correlation coefficient with an ideal ABR waveform (standard template). (**A**) The *RMSE* was calculated to show the deviation of the actual values from the sigmoid function approximation curve. This calculation was performed for each of the ABR waveforms obtained from 13 WT mice. It was found that the *RMSE* increased below a correlation coefficient of 0.60. (**B**) Values with a correlation coefficient < 0.60 showed significantly higher *RMSE*. This result indicates that 0.60 is the optimal reference value for determining whether the waveform is disturbed or not. Data were analyzed using an independent two-sample *t*-test (* *p* < 0.05).

The *RMSE* calculation results suggested that a correlation coefficient of 0.60 is a suitable reference value for discrimination (Figure 2A,B). When the correlation coefficient with the standard template was <0.60, the ABR threshold based on the sigmoid proximity curve was critically different from the threshold or could not be calculated.

**Figure 3:**
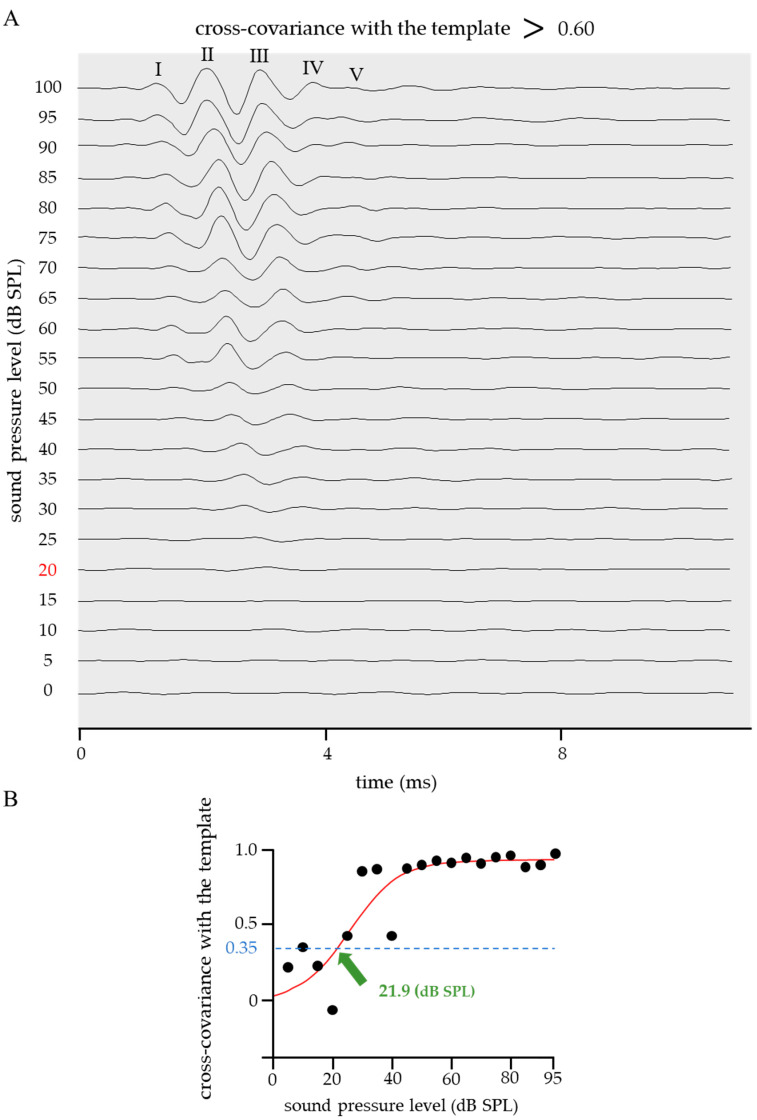
An example of an ABR waveform whose cross-covariance with the template was >0.60. (**A**) When the cross-covariance with the template was >0.60, the ABR waveform had clearly defined waves I–V. The visually determined ABR threshold was 20 dB SPL (red). (**B**) The results of fitting the correlation coefficient vs. sound pressure level with the sigmoid function are shown. The sigmoid function fitting was successful (*RMSE* = 0.16). The ABR threshold was 21.9 dB SPL when the reference value was 0.35, which is close to the visual threshold of 20 dB SPL.

**Figure 4:**
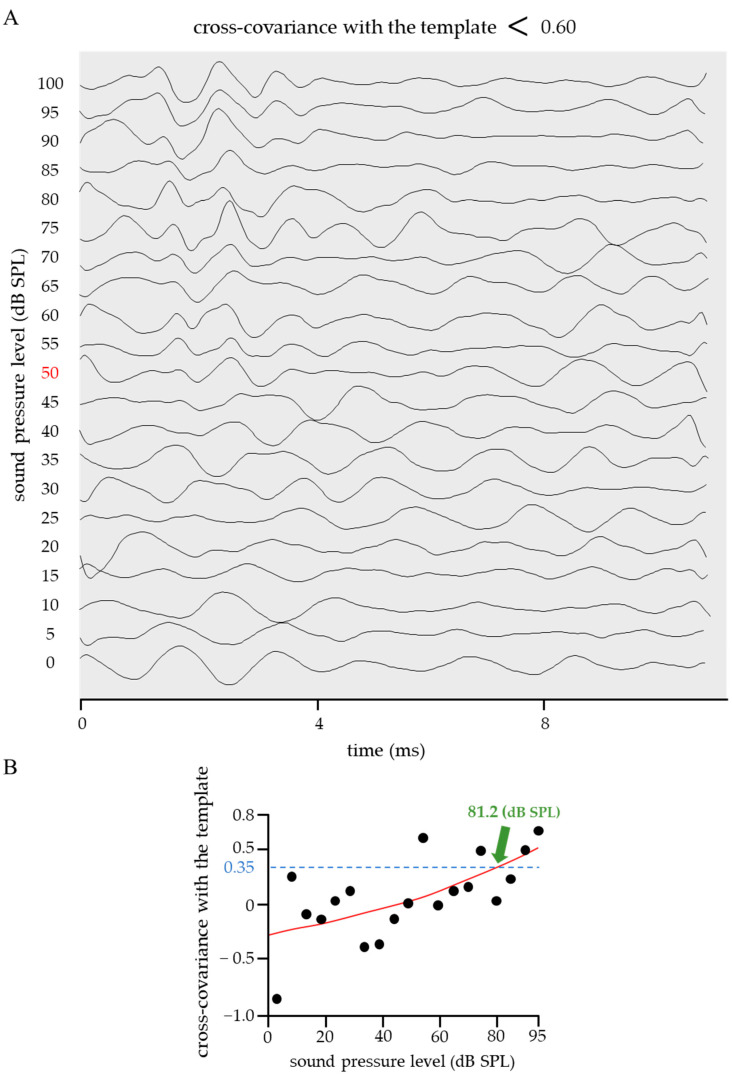
An example of ABR waveforms whose cross-covariance with the template was <0.60. (**A**) When the cross-covariance with the template was <0.60, the ABR waveform lacked clearly defined waves I–V due to high levels of background activity and system noise. The ABR testing was repeated, and the visual threshold was 50 dB SPL (red). (**B**) The results of fitting the correlation coefficient vs. sound pressure level with the sigmoid function are shown. The sigmoid function fitting did not work since the actual values extensively deviated from the approximate curve (*RMSE* = 0.33). The ABR threshold was 81.2 dB SPL when the reference value was 0.35, which is far from the visual threshold of 50 dB SPL.

The cross-covariance between the ideal ABR waveform shown in Figure 1 and the obtained ABR was calculated; further, cases in which the correlation coefficient was above or below 0.60 are shown in Figure 3 and Figure 4. The calculation range for both the cross-covariance with the template and that between adjacent pressure levels represented the entire ABR waveform. When the correlation coefficient > 0.60 (Figure 3), the ABR waveform had clearly defined waves I–V, and the sigmoid function fitting was successful (*RMSE* = 0.16). When the threshold was calculated based on a sigmoidal approximation curve with a correlation coefficient criterion value of 0.35, the value was 21.9 dB SPL, which is close to the visual threshold of 20 dB SPL (Figure 3, red). Contrastingly, in the case of a correlation coefficient < 0.60 (Figure 4), the ABR waveforms lacked clearly defined waves I–V due to high levels of background activity and system noise. Furthermore, the sigmoid function fitting had a large *RMSE* (0.33), which indicates that an accurate approximation was not performed. In the shown example, the ABR threshold based on the sigmoid proximity curve was 81.2 dB SPL, which critically differed from the visual threshold of 50 dB SPL (Figure 4, red).

### 2.3. Improved Quantitative ABR Threshold Algorithm

**Figure 5:**
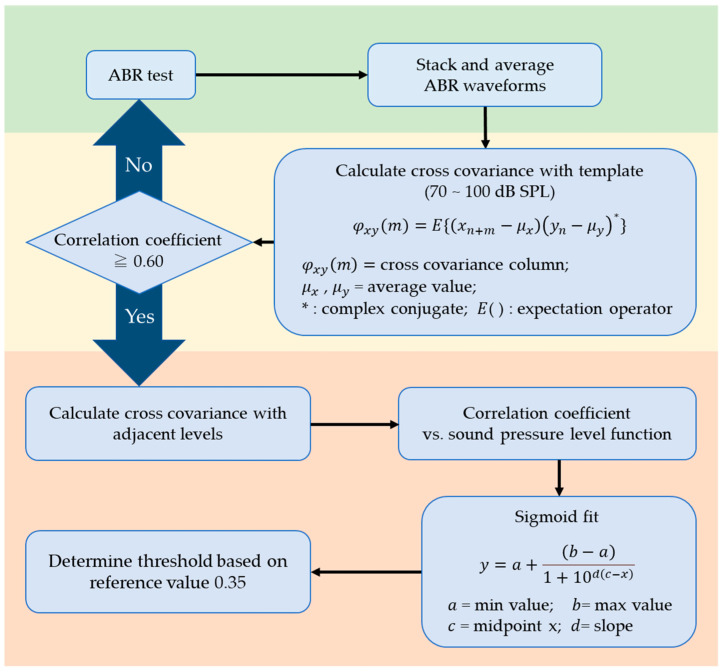
Flowchart for the new ABR thresholding algorithm. Procedures in the yellow layer are those newly proposed in this paper. The input was an ABR waveform level series, which was tacked and averaged (green layer). The cross-covariance with the ideal ABR waveform was calculated. If the correlation coefficient was <0.60, the ABR testing was repeated (yellow layer). If the correlation coefficient was ≥0.60, mutual covariance was calculated for adjacent sound pressure levels to establish the correlation coefficient vs. sound pressure level function. The ABR threshold was determined by fitting with a sigmoid function and setting the reference value at 0.35 (orange layer).

Figure 5 shows a flowchart of the improved quantitative ABR threshold algorithm based on a previous study. The changes from the previous study are as follows:

i. Only perform sigmoid function fitting without fitting by a binomial power function;
ii. The ABR waveform disturbance was determined based on the cross-covariance with the template before and after sigmoid function fitting.

Regarding (i), previous studies fitted sigmoidal functions and binomial power functions, followed by the selection of the function with the smallest *RMSE*. In the audible region, the correlation coefficient value should be constantly high since waves I–V appear in a similar manner; contrastingly, in the inaudible region, the correlation coefficient should be constantly low since only noise exists. Therefore, the approximate curve should theoretically be in the form of a sigmoid function; in this study, we only used the sigmoid function for fitting. Regarding (ii), previous studies required the interphase reference value to range from the minimum *a* and maximum *b* and the slope *d* to be 0.005 < *d* < 0.999. However, there was a clearer difference between audible and inaudible sound pressure for larger d values, with even some examples of values matching the visual threshold, even at 0.999 < *d*. Therefore, the cross-covariance with the ideal ABR waveform (standard template) was calculated prior to sigmoid function fitting. If the mean cross-covariance value is <0.60, the measurement setting may be incorrect, and the results may be unreliable.

In such a case, the ABR measurement was repeated on the same mice, with the aforementioned process being repeated until a correlation coefficient ≥ 0.60 was reached. Accordingly, the number of mice used was minimized.

## 3. Discussion

When assessing auditory function, it is important to establish the threshold of the electrophysiological response “ABR” generated by sound stimulation. Conventionally, the ABR threshold is visually determined by the assessor; however, since the signal-to-noise ratio is low around the ABR threshold, it is easy to make an incorrect decision due to bias. To this point, ABR assessments by clinical experts yield between rater differences in the ABR thresholds of up to 60 dB SPL [5]. Other studies have demonstrated differences in judgment among experienced professionals [13]. Accordingly, various approaches for objectively determining the threshold have been proposed over the past several decades. In 1980, a method was devised for determining the amount of noise in the averaged evoked potential data [14]. This allowed the determination of whether a particular peak component was an auditory signal or a random occurrence. A study comparing the effectiveness of visual and objective methods for detecting ABRs showed higher mean sensitivity scores for methods using correlations than for those using variance ratios and multiple z-test methods [13]. This indicates that the method using correlations is useful for mitigating evaluator bias and ensuring consistency in threshold values. Using correlations as detection criteria, a computer-based algorithm was developed for automatically determining the evoked response thresholds [15]. Other studies have applied cross-correlation functions to evaluate middle latency response (MLR) thresholds [16], human click-evoked brainstem auditory-evoked responses (BAER), and brainstem frequency-following responses (FFR) [17]. The objective thresholding method using cross-correlation functions is among the most favorable methods. A previous study [12], which was the focus of the present study, proposed a method for determining the ABR threshold by calculating the mutual covariance between adjacent sound pressure levels. This study used a previously published dataset and ran the algorithm on ABR waveforms from a wide variety of mice (mice with normal to impaired cochlear function, as well as young to aged mice), which increased the generalizability of the results. The difference between the threshold determined visually and through the algorithm was within 2.9 dB SPL, which suggests that this method does not significantly deviate from conventional visual judgments and that it is reliable and unaffected by bias.

The present study introduced two improvements over this previous method, which yielded a method that was simpler and less burdensome for experimental animals. The first improvement was the approximation of the correlation coefficient vs. sound pressure level function using only a sigmoid function. By excluding the step of approximating the binomial power function, the algorithm user only needs to learn the method of fitting using a sigmoid function, which significantly simplifies the process. This method is further simplified by the exclusion of the requirement to determine the approximate curve based on the *RMSE*. The second improvement was sigmoid function fitting by calculating the mutual covariance with an ideal ABR waveform (standard template) promptly after obtaining the ABR waveform, rather than using a pre-determined criterion after fitting. Accordingly, the step of approximating the ABR waveform with a sigmoid function can be omitted when determining the ABR waveform disturbance, which allows quicker ABR retesting if the fitting is unsuccessful. Accordingly, the overall time for ABR measurement is reduced, which minimizes the burden on the experimental animals. The template was created by averaging the ABR waveforms that were successfully approximated using a sigmoid function. In this study, template ABR waveforms were only obtained from WT mice. However, templates could be established for each group as needed in order to adapt to differences in latency and waveforms depending on the pathology of the mouse and the measurement environment. This method is not a burden on the user by the fact that only seven sound pressure levels of 70–100 dB SPL need to be averaged. The threshold value of 0.60 was determined based on 13 ABR waveforms. However, only three ABR waveforms were determined to be unsuitable for sigmoid function fitting due to large *RMSE*s. This could be attributed to the small frequency at which the disturbed ABR waveforms were obtained. Further ABR measurements will be performed to increase the number of samples to allow more accurate threshold determination. Furthermore, the present method can be applied to other kinds of mammal animals by obtaining the reference ABR template for each model animal in principle. The clinical test of AABR (Automated Auditory Brainstem Response) in human newborn hearing screening automatically determines whether the patient has a hearing loss (pass or refer) in a specific sound pressure of 35 dB SPL. On the other hand, this study demonstrated the quantitative criteria for determining the ABR threshold using a standard template to determine whether the ABR waveform is disturbed.

Taken together, the improvements presented in this paper yielded an objective method for threshold determination using cross-correlation functions that can be used to easily analyze auditory functions in various pathological conditions. In research aimed at understanding and overcoming human diseases, laboratory animals such as mice and rats are generally used [18,19,20,21,22,23]. This is also true for diseases associated with hearing loss. Analysis of diseased animals, including genetically engineered animals, such as transgenic mice and knockout mice, is essential to clarify the causes and pathogenic mechanisms of hearing impairment in humans [24,25,26]. Furthermore, in order to resolve the underlying causes of such hearing loss, it is necessary to focus on the transmission of sound waves in the ear ossicles and cochlea and to investigate the causes from the cellular level [27]. Not only bone mineral density but also bone microstructure can be a key regulator in determining hearing properties [28]. Specifically, the microarrangement of collagen/apatite in auditory ossicles is reported to be associated with hearing functions. It is also important to develop devices to overcome hearing loss aiming at treatment from the cellular and tissue level [29]. Our improved quantitative ABR threshold determination method can determine the impact of the disease on auditory function in a simple and bias-free manner.

## 4. Materials and Methods

### 4.1. Model Mouse

This study was conducted using 5-week-old C57BL/6 mice. Animal experiments were approved by the Animal Experiment Committee of the Graduate School of Engineering, Osaka University (Approval No. 2021-02-0). Mice were housed in a facility with constant humidity and temperature and a 12 h light–dark cycle.

### 4.2. ABR Testing

ABR testing device was composed of RZ6 Multi I/O Processor, Medusa4Z Amplifier, and MF1 Multi-Field Magnetic Speakers as hardware and BioSigRZ version 5. 7. 5 as software. All were manufactured by Tucker–Davis Technologies (Alachua, FL, USA).

The experimental mice were first weighed, followed by intraperitoneal administration of 0.01 mL/g of three types of mixed anesthetic agents. Triple anesthesia was induced using a mixture of 1 mg/mL medetomidine hydrochloride, 5 mg/mL midazolam, 5 mg/mL butorphanol tartrate, and saline. After confirming that there was no response to touch stimuli, eye ointment was applied to both eyes to remove the noise effects resulting from the blink reflex, and the mice were moved into a soundproof room. A non-electric heating pad (which did not generate electrical signal noise) wrapped in aluminum foil was placed in the soundproof room to prevent the mice from losing body temperature, and the mice were placed in a prone position. The ground electrode was connected to the aluminum foil, followed by the insertion of two recording electrodes with needle tips and one reference electrode at three locations on the head. Specifically, the recording electrode was located under the right and left auricularis, while the reference electrode was located at the top of the head, such that the needle and subcutaneous portion extensively made contact to reduce electrical resistance. After confirming that the resistance of the electrodes was <1.5 kΩ, a tube connected to a speaker was inserted into the left ear. Next, the soundproof room was closed, and testing began. Tone burst sounds were used as sound stimuli. The tone burst sounds comprised a sine wave that included rising and falling portions. Testing was performed at a 16-kHz frequency with a duration of 1 ms and a rise and fall time of 0.1 ms each. For each sound stimulus, the ABR was calculated by decreasing the sound pressure level from 100 dB SPL to 5 dB SPL at 5 dB SPL intervals. Since the ABR for a single sound stimulus was weak, we performed an additive averaging process 500 times at each sound pressure level. Finally, the ABRs were processed with a 500 Hz high-pass filter and a 3000 Hz low-pass filter to remove noise.

### 4.3. Quantitative Determination of ABR Threshold

Using the ABR waveforms obtained through the aforementioned methods, the ABR threshold was objectively determined using the numerical analysis software MATLAB R2022b (MathWorks), as described previously [12]. Specifically, the mutual covariance was first calculated by MATLAB with a one-step higher sound pressure level. Mutual covariance was the degree of similarity between two data with a time difference (Lag). The mutual covariance was plotted against the sound pressure level in 5 dB SPL intervals from 95 dB SPL, and the graph was fitted with a sigmoid function. The ABR threshold was defined as the sound pressure at which the cross-covariance was 0.35 in the approximate curve. The value of 0.35 was determined based on the previous report [12], which clarified the threshold determinations algorithm considering the measurement differences among observers.

This allowed quantitative determination of the ABR threshold; however, sigmoid function fitting was difficult for some waveforms obtained by ABR testing. Therefore, we established an ideal ABR waveform (Figure 1) and calculated the cross-covariance between the template and the obtained ABR waveform. The analysis range was 70–100 dB SPL (in 5 dB SPL intervals). The calculation range for both the cross-covariance with the template and that between adjacent pressure levels represented the entire ABR waveform. When the correlation coefficient was >0.60, the ABR waveform showed clearly defined waves of I–V, and was successfully fitted with the sigmoid function (*RMSE* = 0.16). A mean cross-covariance value < 0.60 suggested incorrect measurement settings and unreliable results; therefore, the ABR test was repeated on the same mouse.

## Data Availability

The data presented in this study are available upon reasonable request from the corresponding author.
